# Problematic mental health-related advice: exploring the role and limitations of EU's digital toolbox on the case of #TherapyTok

**DOI:** 10.3389/fpubh.2026.1793306

**Published:** 2026-05-01

**Authors:** Tjaša Petročnik

**Affiliations:** Tilburg Institute for Law, Technology, and Society, Tilburg University, Tilburg, Netherlands

**Keywords:** Digital Services Act, misinformation, public health prevention, social media, TherapyTok

## Abstract

Social media platforms are increasingly becoming resources for mental health advice and validation. This might contribute to awareness-raising and promoting healthy behaviors, but it also raises concerns due to proliferation of misinformation and unproven claims. The brief assesses EU's frameworks that aim to ensure online environments are safe, including the DSA, the announced DFA, and other consumer protection regimes. Applying the public health prevention framework by Ishizumi et al., the brief argues for a public health-informed approach to counter the identified #TherapyTok concerns. For example, EU digital governance frameworks like the DSA should explicitly include public health evaluation criteria in enforcement. The brief offers further EU policy recommendations, proposing more decisive action might be needed especially in upstream prevention.

## 1 Introduction

For the past two decades, social media platforms have enabled users to create, share, and consume content and engage with other users (1). With their ability to spread information and connect people, including on mental health matters, Instagram and TikTok are fast becoming “the psychiatrist couch of Gen Z” (2). The phenomenon of #TherapyTok—influencers offering advice or self-care strategies, sharing own mental health struggles and tips, explaining psychological concepts or discussing ‘hot takes' about therapy (3–7)—shows a novel way of engaging with digital technologies for mental health.

The low access barrier to such content has both potential benefits and drawbacks. While #TherapyTok, here used to refer to mental health influencers and content across social media platforms in general beyond just TikTok (8), could be a positive development—it might namely contribute to awareness raising, promoting healthier behaviors, and empower individuals with advice, validation or support (3–6, 9–12) –, it likewise raises concerns. These are linked to problematic social media use, the worsening of negative emotional and psychological symptoms or behaviors (13), as well as to “peddling misinformation, including misused therapeutic language, ‘quick fix' solutions and false claims” (14). When relying on the latter for informing one's health attitudes or decisions, mental health and wellbeing of those that consume this content could be negatively affected, making this phenomenon a considerable public health risk.

Against this background, this policy brief examines EU's regime for regulating social media, especially the frameworks in place for ensuring safe digital environment and protecting individuals from, among other things, harms to (mental) health. Specifically, it assesses the capacity of EU's platform governance instruments for tackling the risks of potentially misleading, inappropriate, or otherwise harmful mental health information proliferating on social media. For this, the paper discusses the role and limitations of the selected EU instruments through the lens of the public health prevention framework for managing informational ecosystems as developed by Ishizumi and colleagues (15), and proposes policy recommendations for a more comprehensive response to the identified risks.

Whereas the public health prevention framework was developed in the context of Covid-19 and the ensuing pandemic of misinformation (15), here, its relevance is showcased also for the #TherapyTok harms by utilizing it as an evaluative framework to examine and inform EU's regulatory approach to the growing use of social media platforms. The central proposition of this brief is that the EU approach exhibits significant potential to address the supply side of #TherapyTok content, as EU digital market regulation instruments can be effectively used to address harmful platform design and functioning that contributes to spread of misinformation downstream and intervene against misleading or unsupported health claims. Yet, from a public health perspective on ‘infodemics', this is insufficient, since public health threats also emerge from how people interact with social media information and why (15). A more comprehensive approach would thus require engagement also with the demand side and the context of content consumption. There, the current role of the EU is limited, which in turn calls for greater policy attention also for upstream prevention, bringing mental health determinants more expressly under the auspices of digital governance.

## 2 Platformization of mental health and potentially harmful effects of social media content

Various risks can be associated with consuming mental health-related content on social media platforms. For one, there is content that can be actively harmful or disturbing. This might include users being repeatedly exposed to content that for example promotes self-harm or eating disorders, or content that is associated with unhealthy behaviors (16, 17). However, even content that aims to be, or poses as, helpful, can be problematic. Such content is the focus of the present paper.

Firstly, #TherapyTok is rife with relatable and often humorous videos made by both therapists and individuals with lived experience. But not all such content is correct; it has been reported that around 80% of #mentalhealthadvice on TikTok could be considered misleading and one third scientifically inaccurate (11, 18). According to an investigation by The Guardian, half of the top #mentalhealthtips videos offering advice on trauma, neurodivergence, anxiety or depression contains misinformation. But even if accurate, the advice can be misinterpreted or misapplied. Such short-form content also tends to generalize and leave little space for nuance (14), making it less applicable to specific individual's context or needs.

Secondly, the format of social media makes it difficult to ascertain the influencer's expertise and credibility (7, 9, 19) and the content often lacks disclaimers that the creator is not qualified to give mental health advice (20). As a consequence, consumers of such content might place their trust in dubious or unsuitable sources of information (21, 22).

Thirdly, there is a risk of conflict of interest. While influencers enjoy a bond with their followers that tend to trust their recommendations, content creators might also have an incentive to use their personal brand and platform for financial or other gains, for instance by promoting (unproven) treatments or omitting sponsorship disclosures (14, 23), casting doubts over some influencers' underlying motivations.

These risks are further compounded by the nature of social media platforms, where more controversial claims have a higher probability of going ‘viral' and where behavior predictions are employed and ‘echo chambers' and ‘rabbit holes' occur (5, 13). This can further exacerbate harmful effects or individual vulnerabilities, not only due to repeated (algorithmic) exposure to such content, but also thanks to platforms' business model that tends to aim for maximum user engagement (3, 24).

When materialized, these risks might then have serious and tangible consequences for mental health and wellbeing. For instance, reliance on misconceptions or inappropriate or dubious advice might worsen psychological symptoms, lead to wrongful self-diagnoses or employing useless or unproven coping strategies, and preclude individuals from seeking (professional) help or, conversely, pathologize otherwise normal human emotions (5, 14).

From a public health perspective, high-quality social media content is desirable as it can effectively support individuals in preventative health behaviors. While non-quality content is obviously not, since it can be detrimental to mental health of those consuming it, it might still be rewarding from the perspective of content creators or platforms. The disincentive to limit the proliferation of such content makes policy and regulatory action justified, in particular in a context where people increasingly struggle with mental health and care is not always readily accessible (25). The EU has already taken action in this regard, as demonstrated in the next section.

## 3 EU's toolbox for regulating social media content

Recently, the EU undertook big steps toward a more decisive governance of digital platforms, recognizing that while they can act as key avenues for accessing information, their activity might also have significant societal implications (26, 27). Most notably, the Digital Services Act (DSA) introduces accountability and transparency measures for online intermediaries, putting forward a tiered approach to obligations for the providers of these services, including online platforms.

In short, the DSA requires the providers of online platforms to have systems in place to remove illegal content, to be transparent about their content moderation practices and put in place procedural safeguards that protect users in relation to content moderation, to provide information about content recommendation algorithms and advertising, to refrain from designing their online interfaces in a deceptive or manipulative way, and to protect the privacy, safety, and security of their users that are minors (27, 28). Very large online platforms (VLOPs) like Instagram and TikTok (29) are subject to additional stringent obligations, outlined in Sec. 5, Ch. III DSA. These include *ex ante* duties to assess and mitigate systemic risks to, among others, public health and and users' (mental) wellbeing stemming from content amplification or platforms' algorithms. They also include the requirement to open platform data to researchers studying these systemic risks.

In addition to the DSA, European Commission announced the Digital Fairness Act (DFA) with the aim of modernizing EU consumer protection rules for online interactions. This regulatory update, the proposal for which is expected in 2026, is considered necessary because consumers generally feel a lack of control over their online experiences, for instance due to ‘dark patterns', addictive design, personalized targeting or lack of disclosures in influencer marketing (30). With this, the DFA is poised to address these concerns and the very architecture of online environments that commonly prioritizes platform's interests over user benefit (31), and could be another major step toward ensuring healthier online environments.

EU rules also concern the activities of social media influencers, in particular when they monetize their content and act as advertisers or sellers, that is, promote their own products or services or those of others (32). Here, existing consumer protection rules, including for example the Unfair Commercial Practices Directive (UCPD) and the Consumer Rights Directive (CRD), ensure protection from misleading or aggressive commercial practices or unsupported health claims, mandate information that needs to be provided prior to concluding a contract, and might offer remedies to offset damages suffered due to ‘snake oil' transactions (28).

In sum, EU's toolbox concerning harmful social media content related to mental health, or otherwise, is far from empty. In this regard, EU frameworks like the DSA could be significant, especially concerning the design and functioning of digital platforms, since they are built on the understanding that these actors can cause considerable societal harm. In this light, they to an extent align with typical public health interventions aimed at ensuring product safety and autonomy and information in consumers' health decision-making (31).

Moreover, while the DSA does not *per se* prohibit the sharing of harmful content or misinformation, including related to (mental) health, that does not constitute illegal content, it does acknowledge that the spread of such information nonetheless has risks and thus places enhanced responsibilities on private providers of VLOPs due to their structural position (33). This might indeed include measures that restrict the dissemination and reach of misinformation. A caveat here is that VLOPs have, in identifying and mitigating these systemic risks, considerable discretion (33, 34). Whereas this seems a deliberate choice of the legislator, these entities primarily have system and interface development expertise and are not public health experts, meaning that their envisioned measures might not be fully adequate from a public health perspective.

Further, while consumer protection rules are noteworthy, since they afford protection to social media users from misleading claims or practices, they only apply insofar as content creators monetize their content and thus bear less relevance when influencers merely share their opinions, lived experience or advice without selling anything. Lastly, the DFA reform of the consumer protection rules for the online environments might serve to uphold public health by addressing harmful design choices and online architectures, but its potential, that is, the scope and ability to meaningfully address problematic online practices cannot be evaluated before the proposal is made public.

## 4 The limitations of EU's economic governance for public health approach to social media through the public health prevention viewpoint

The interim conclusion here is that there is considerable merit to using EU's market and platform regulation role, including from a public health viewpoint, against the harms of #TherapyTok. Yet, as this next section discusses, viewing EU's regulatory action through the lens of the public health prevention framework (15) indicates that the current approach has limitations.

From the previous analysis, it namely becomes evident that the discussed frameworks primarily affect the production and distribution of social media content, that is, what content consumers are exposed to (35). This focus on the supply side corresponds to research that suggests digital platforms should better mitigate risks associated with recommending and spreading problematic content and provide users with more effective control over what they see (13). It is thus commendable that the EU is considering measures that place the onus of policy debates on the role of social media platforms and their business model (36), thus utilizing its technical and market regulation powers. But, as it will become clear next, this approach is in itself incomplete, since threats to public health are not only driven by the (supply of) suboptimal quality of online information downstream; they are also determined by the context of both the supply, but, perhaps more importantly, the demand for information (15), considerably expanding the scope of the required regulatory and policy action.

As posited, in addition to quantity and quality of the content, public health can also be affected by how and why people consume, produce, interact with, and behave around information (15). This in turn requires a more comprehensive response that extends beyond measures reacting to misinformation and necessitates focus also upstream [ibid.]. In other words, the onus of policy interventions also needs to be placed on the societal context that affects the demand and allows such technological developments to proliferate, which the EU's current digital regulation approach currently largely leaves unaddressed.

In this sense, to manage information ecosystems holistically, Ishizumi and colleagues propose a public health prevention framework that encompasses four levels of prevention and both down- and upstream prevention strategies: primordial (tier 1), primary (tier 2), secondary (tier 3), and tertiary (tier 4) prevention. These levels span different policy actions and interventions, ranging from strengthening health systems and generating high-quality evidence (1); improving digital literacy and inoculating individuals against misinformation techniques (2); detecting trends in the spread of harmful content and saturating the information ecosystem with high-quality information (3); and acute responses like fact-checking, public information campaigns, and platform-based friction interventions, including content moderation or information disclosures (4) [ibid.].

When examining the above-addressed EU frameworks from this view, it becomes evident that they mainly contribute to secondary and tertiary prevention (tiers 3 and 4), focusing on ensuing access to data for identifying public health threats, equipping consumers with informational cues to make sense of their online interactions, and introducing transparency and friction in harmful content supply. In itself, this is not surprising, since constitutionally, the EU has a very pronounced market regulating role, but relatively weaker competences regarding public health and welfare interventions (37) that are arguably more in line with upstream prevention levels. Yet, from the evaluative perspective offered by the public health prevention framework, such centring of economic governance measures that are oriented downstream, that is, on addressing the spread of problematic content itself, risks overlooking other factors that impact mental health, drive people to social media content in the first place, and determine how they engage with it. The public health prevention framework thus allows us to bring to fore also the need for strategies that engage with structural determinants of ‘infodemics' (15, 21), that are currently not evident in the analyzed toolbox. The next section thus offers some corresponding recommendations for EU policy to close the loop on harms from #TherapyTok.

## 5 Policy recommendations

Firstly, while the responsibilization of platforms under the DSA and, likely, the DFA is a step in the right direction, the devil, as usual, hides in implementation and enforcement. Here, it needs to be ensured the DSA's potential is aligned with public health requirements. In terms of successful tertiary prevention, the European Commission could, in line with Art. 35 DSA, develop policy guidance with concrete benchmarks and best practices for content moderation, boosting high-quality content, and auditing algorithms specifically tailored to issues related to (mental) health and how it might be impacted by social media content. It could also use its power to facilitate the development of codes of conduct on these matters, as enabled by Art. 45 DSA, making sure that all relevant stakeholders are included and heard in the process. Most importantly, when evaluating the systemic risk assessment and mitigation reports, the Commission should consider, if possible consulting relevant sectoral units or authorities, the suitability of the measures through a public health lens and decisively use its powers of investigation and enforcement concerning the spread of and deplatforming problematic (mental) health content (34, 38), should the risks materialize in a sufficiently serious manner.

Further, stricter enforcement regarding advertising and disclosures and protection from false claims might be needed under consumer law, and options investigated for more consistent implementation of informational cues and accuracy and trustworthiness signifiers (33, 39) across platforms and diverse mental health issues. Again cooperation between consumer and public health authorities and stakeholders might be called for. It might also be sensible to explore whether platforms might be incentivized to impose limits on content monetization when influencers share problematic mental health content or promote unsupported products, although this might endow platforms with additional power, which might be undesirable (28). Relatedly, but beyond the scope of this brief, professional bodies that oversee the work of mental healthcare providers could provide guidance on social media use by mental health professionals (7) when they act as content creators.

As the public health prevention model indicates, however, these activities—while undoubtedly important—represent only a partial solution. As the Commission undertakes its inquiry on the broader impacts of social media on wellbeing and mental health (40), it is therefore imperative that its scope extends beyond downstream strategies that focus on platform design, content distribution, transparency, and user control. However, what is known about the inquiry from publicly available information, seems inconclusive, as the exercise aims to “build on enforcement actions currently under way in the context of the Digital Services Act” against Meta and TikTok (41). While major developments for mental health, these are primarily concerned with social media interfaces and recommender systems that might exploit young people's vulnerabilities and lead to behavioral addictions (42, 43), and thus arguably primarily consider platform friction countermeasures concerning infinite scrolling and use breaks.

The inquiry is framed as a follow-up to Commission's Communication on a comprehensive approach to mental health (44). Therein it is acknowledged that digital tools can affect mental health both positively and negatively. A healthier digital space, especially for the youth, would thus include not only protection from problematic content or aggressive marketing, and online environments that support, or at least do not actively undermine mental health and wellbeing, but also safeguards against excessive screentime and promoting prudent social media use among others (45). It would therefore indeed be advisable that the social media inquiry orients its scope, and possible subsequent policy intervention, to the breadth of this Communication, of course in line with EU's existing public health competences.

In this light, and secondly, a stronger focus on primary and secondary prevention that would more proactively engage with the demand-side is needed. This could include facilitating literacy measures to address problematic social media use and enhance critical thinking skills and emotional competences (1, 5, 13, 36, 46). As is further discussed below, empowering individuals is paramount, but the onus should not only be placed on them and their responsible use of social media (47). A more pronounced focus upstream might also necessitate exploring the need and options for supporting entities identifying misinformation, facilitating strategic beneficial content generation and outreach work by socio-pedagogical specialists in online spaces to identify and debunk misinformation and channel individuals toward reliable sources of support and information, akin to ‘street work' (5, 38, 48), as well as equipping teachers, families or other ‘real life' authorities with helpful resources (38, 49).

Thirdly, the attention of the inquiry and ensuing policy activity could be more decisively placed on primordial risk and prevention factors like population resilience (15) and socio-economic conditions that might increase vulnerability to harmful mental health content, as far as this is constitutionally permissible. Namely, sociodemographic factors and inequalities might impact individuals' susceptibility to health misinformation or exacerbate problematic social media use, and health misinformation might not affect everyone equally (38, 39, 50). Regarding possible actions, the EU could, in terms of such upstream prevention, support Member States with sharing best practices and dedicated funding for research and targeted interventions in this area.

## 6 Conclusion

Mental health does not “depend on healthcare services alone but also on the health of the informational environment” (51). Here, social media has considerable potential to contribute to public health prevention, but also to affect it negatively (38). In this light, the EU's aspiration to impose heightened responsibility for the spread of misinformation or otherwise harmful information on platforms is only welcome. Yet, relying on the public health prevention model (15), this paper urges EU policymakers to direct their attention also upstream and explore, to the extent constitutionally possible, in what way might the EU manage the ‘infosphere' as a social determinant of health (21). This means that policy strategies should not only consider the supply side of mental health-related social media content, but also tackle its consumption and the reasons why people, especially the youth, engage with it. In this sense, the dangers of #TherapyTok should be approached as symptoms of a wider problem. Taking this problem seriously then invites a stronger responsibility for the EU, as well as its Member States, to ensure meaningful empowerment of social media users and engage with socioeconomic disparities, and the way in which these relate to consumption of social media content.
